# D1 receptor hypersensitivity in mice with low striatal D2 receptors facilitates select cocaine behaviors

**DOI:** 10.1038/s41386-018-0286-3

**Published:** 2018-12-01

**Authors:** Lauren K. Dobbs, Alanna R. Kaplan, Roland Bock, Khanhky Phamluong, J. Hoon Shin, Miriam E. Bocarsly, Lindsay Eberhart, Dorit Ron, Veronica A. Alvarez

**Affiliations:** 1 Laboratory on the Neurobiology of Compulsive Behaviors, National Institute on Alcohol Abuse and Alcoholism, Intramural Research Program, NIH, Bethesda, MD USA; 2 Center on Compulsive Behaviors, Intramural Research Program, NIH, Bethesda, MD USA; 3 Department of Neurology, University of California San Francisco, San Francisco, CA USA; 4 Postdoctoral Research Associate Program, National Institute of General Medical Sciences, NIH, Bethesda, MD USA; 5 National Institute on Drug Abuse, Intramural Research Program, NIH, Baltimore, MD USA

**Keywords:** Addiction, Cellular neuroscience

## Abstract

Vulnerability for cocaine abuse in humans is associated with low dopamine D2 receptor (D2R) availability in the striatum. The mechanisms driving this vulnerability are poorly understood. In this study, we found that downregulating D2R expression selectively in striatal indirect-pathway neurons triggers a multitude of changes in D1 receptor (D1R)-expressing direct-pathway neurons, which comprise the other main subpopulation of striatal projection neurons. These changes include a leftward shift in the dose-response to a D1-like agonist that indicates a behavioral D1R hypersensitivity, a shift from PKA to ERK intracellular signaling cascades upon D1R activation, and a reduction in the density of bridging collaterals from D1R-expressing neurons to pallidal areas. We hypothesize that the D1R hypersensitivity underlies abuse vulnerability by facilitating the behavioral responses to repeated cocaine, such as locomotor sensitization and drug self-administration. We found evidence that littermate control mice develop D1R hypersensitivity after they are sensitized to cocaine. Indeed, D1-like agonist and cocaine cross-sensitize in control littermates and this effect was potentiated in mice lacking striatal D2Rs from indirect-pathway neurons. To our surprise, mice with low striatal D2Rs acquired cocaine self-administration similarly to littermate controls and showed no significant change in motivation to take cocaine but lower seeking. These findings indicate that downregulation of striatal D2Rs triggers D1R hypersensitivity to facilitate cocaine locomotor sensitization, which by itself was not associated with greater cocaine taking or seeking under the conditions tested.

## Introduction

Cocaine abuse and dependence is a prevalent public health concern marked by compulsive drug seeking and taking. Over the past several decades, significant advancements have been made in elucidating the mechanisms of cocaine reward and locomotor activation, which are associated with acute increases in striatal dopamine transmission resulting from cocaine blocking the dopamine transporter. These actions of cocaine are well characterized in vivo and in vitro in animal models and humans [1–5]. However, the mechanisms driving the vulnerability to cocaine abuse have been difficult to identify, and the underlying substrates remain unclear.

A large body of preclinical and clinical studies indicates that low dopamine D2 receptor (D2R) expression within the striatum is associated with cocaine abuse. For instance, a landmark PET imaging study found reduced D2R availability in the striatum of individuals with a history of cocaine abuse compared to healthy controls [6]. Follow-up investigations found that decreased striatal D2R availability is associated with greater cue-induced cocaine craving in humans and larger cocaine place preference in rats [7, 8]. Data from non-human primates [9] and rats [10] also indicate that pre-existing low levels of striatal D2Rs predispose subjects to take more cocaine than subjects with higher D2Rs. Rats with low D2Rs that self-administered more cocaine exhibited greater trait-impulsivity, a behavioral phenotype associated with compulsive drug seeking and taking [10]. Conversely, overexpression of striatal D2Rs reduced cocaine self-administration in rats [11].

Pharmacological and genetic approaches in animal models support the hypothesis that low striatal D2R availability and function drive the vulnerability to cocaine abuse. However, the mechanisms are still unknown, and it is unclear which subpopulation of striatal D2Rs is important for driving the vulnerable phenotype. Within the striatum, D2Rs are expressed on multiple cell types: in approximately half of the striatal projection neurons, which are referred to as indirect-pathway medium spiny neurons (iMSNs), in cholinergic interneurons, and in axonal projections from midbrain dopamine neurons and cortical and thalamic inputs to the striatum.

We recently identified a specific subset of striatal D2Rs that are likely mediating this vulnerability. Deletion of D2Rs selectively from iMSNs facilitated the acquisition of cocaine place preference and expression of locomotor sensitization to repeated cocaine exposure, despite severely blunting the acute cocaine locomotor response [12, 48]. These data suggest that reduction of D2Rs selectively from iMSNs can facilitate the behavioral plasticity associated with repeated cocaine administration to enhance cocaine reward.

Striatal D1Rs are expressed in the other half of MSNs that form the direct-projection pathway out of the striatum (dMSNs) and are thought be to necessary for cocaine locomotor sensitization and cocaine reward. Global knockout of D1Rs abolished cocaine self-administration ([13], but see ref. [14]), and administration of a D1-like receptor agonist-induced a place preference [15]. Additionally, the expression of D1Rs in the nucleus accumbens (NAc) was necessary and sufficient for the expression of cocaine locomotor sensitization [16–18], and the mixed D1/D2-like receptor agonist apomorphine cross-sensitized with the locomotor response to cocaine [19]. D1R-expressing dMSNs and D2R-expressing iMSNs interact via an extensive network of GABAergic intra-striatal axon collaterals, which provides lateral inhibition among MSNs. We recently reported that activation of D2Rs in iMSNs inhibits collateral GABA transmission to suppress the lateral inhibition and gate action potential firing of dMSNs [12].

In this study, we investigated striatal D1R sensitivity as a factor driving facilitated cocaine locomotor sensitization downstream of selective D2R reduction in iMSNs, and tested whether this targeted D2R reduction is sufficient to enhance cocaine self-administration. We found that selective D2R deletion from iMSNs enhances striatal D1R signaling and function and is associated with facilitation of cocaine locomotor sensitization. Gross anatomical analysis also provides evidence of a long-lasting structural rearrangement in direct-pathway projections to the pallidum, known as bridging collaterals. We hypothesized that this heightened D1R functionality could subsequently drive greater cocaine intake, however we found no increase in cocaine self-administration rate under the conditions tested here.

## Methods

### Animals

Experiments were performed in accordance with guidelines from the National Institute on Alcohol Abuse and Alcoholism’s Animal Care and Use Committee. Male and female mice (8–25 weeks) were used for all studies. Heterozygous (iMSN-Drd2HET) and homozygous (iMSN-Drd2KO) mice were generated by crossing *Drd2*^*loxP/loxP*^ and *Adora2a-Cre* mice, as previously described [12, 20]. For immunohistochemistry, iMSN-Drd2KO and *Drd2*^*loxP/loxP*^ mice were crossed with mice expressing tdTomato under the *Drd1a* promotor to label dMSNs ([21]; see Supplemental Methods for details). Mice were group housed, except for those undergoing cocaine self-administration, and maintained under a 12:12 h light cycle (6:30 ON/18:30 OFF) with food and water ad libitum.

### Surgical procedures

#### Stereotaxic viral injection

Mice were placed in a stereotaxic frame under isoflurane anesthesia and bilaterally infused with a Cre-dependent viral vector expressing channelrhodopsin-2 (300 nL/side of rAAV5-EF1-DIO-hChR2(H134R)-EYFP; 4.5 × 10^12^, UNC) into the NAc core (see Supplemental Methods). Electrophysiology experiments were performed >2 weeks after surgery.

#### Intra-jugular catheter placement

Male and female iMSN-Drd2HET and *Drd*^*wt/loxP*^ littermate controls were surgically implanted with indwelling jugular catheters (CamCath) as previously described [22]. Catheters were flushed daily with saline, and patency was determined before beginning the experiment (details in Supplemental Methods).

### Drugs

Sulpiride and SKF-81297 (Tocris), cocaine HCl (National Institute on Drug Abuse), ketamine-xylazine and pentobarbital (FLAC facility veterinarian) were dissolved in saline. Intraperitoneal (i.p.) drug administration was delivered at 10 ml kg^−1^ body weight. Intravenous cocaine was delivered based on mouse weight (see below).

### Quantitative polymerase chain reaction

Mice were anesthetized with pentobarbital and decapitated. The brain was removed and the striatum was dissected on ice, homogenized, and RNA was purified using RNeasy Plus Mini kit (Qiagen). cDNA was synthesized using iScript Reverse Transcription Supermix (Biorad). Relative dopamine D3 receptor and beta-actin mRNA expression were determined with TaqMan Gene Expression Assays (Life Technologies) using a StepOnePlus Real-Time PCR system (Applied Biosystems). Relative D3 receptor expression was calculated using the ΔΔCt method. See Supplemental Methods for details.

### Western blot analysis

Mice were anesthetized with pentobarbital and the dorsal medial striatum (DMS) and NAc were rapidly dissected on ice. Tissue was homogenized in 300 µL RadioImmuno Precipitation Assay buffer and homogenates (30 µg) were resolved on NuPAGE Bis-Tris gels (Life Technologies) and transferred onto a nitrocellulose membrane (EMD Millipore). Blots were blocked (5% milk-PBS, 0.1% Tween) for 20 min and incubated overnight with primary antibodies at 4 °C. Membranes were washed and incubated with secondary horseradish peroxidase-conjugated antibodies for 2 h at RT. Membranes were visualized using Enhanced Chemiluminescence Plus (GE Healthcare) and bands were quantified using ImageJ. See Supplemental Methods for details.

### Immunohistochemistry

*Drd2*^*loxP/loxP*^ and iMSN-Drd2KO mice expressing tdTomato under the *Drd1a* promoter were treated with saline or SKF-81297 (5 mg/kg, i.p.; 3 mice/drug/genotype) 15 min prior to transcardial perfusion with 4% paraformaldehyde. Brains were post-fixed and cryoprotected (30% sucrose in 0.1 M PB) overnight at 4 °C. Coronal sections (40 µm) from each drug and genotype group were processed in parallel. Sections were washed in PBS (3 × 10 min) and blocked with 5% goat serum (Vector) for 1 h at RT. Sections were incubated in the primary, rabbit anti-pERK1/2, (72 h at 4 °C), washed in PBS (6 × 10 min), then incubated in the secondary, goat anti-rabbit Alexa-488, (2 h at RT) and washed with PBS (3 × 10 min) and 0.1 M PB (2 × 10 min). Sections were mounted with Vectashield-DAPI and analyzed with confocal imaging (see Supplemental Methods for details).

### Acute D1-like receptor agonist locomotion and cocaine cross-sensitization

Naive, male and female iMSN-Drd2KO and *Drd2*^*loxP/loxP*^ littermate controls were habituated to handling and injection (saline, 10 ml/kg) over 3 days. For the SKF-81297 dose-response, escalating doses were administered (1, 2.5, and 5 mg/kg) with two days washout between each dose. Baseline locomotor activity was monitored for 1 h before saline or SKF-81297 injection to attenuate the novelty-induced dopamine response [23], and activity was recorded 1 h post injection. For the cross-sensitization experiments, separate groups of mice received five consecutive days of saline, SKF-81297 (5 mg/kg), or cocaine (15 mg/kg). Fourteen days after the last injection mice were challenged with either cocaine (15 mg/kg) or escalating doses of SKF-81297 (1, 2.5, and 5 mg/kg), with 2 days between each SKF-81297 dose.

### Operant intravenous cocaine self-administration

Self-administration experiments were performed during the dark phase of the light cycle in modified operant boxes as previously described ([22]; see Supplemental Methods for details). Data were collected by MedPC software and analyzed using Prism (GraphPad) and COBAI, a custom written software package for IgorPro (Wavemetrics) [24, 25].

#### Acquisition

Experiment-naive mice (Males: *Drd2*^*loxp/wt*^, *n* = 9, iMSN-Drd2HET, *n* = 5; Females: *Drd2*^*loxp/wt*^, *n* = 9, iMSN-Drd2HET, *n* = 11) were trained to press a lever on a fixed ratio 1 (FR1) to receive intravenous cocaine over 10 days. Each cocaine infusion (1 mg/kg, 12–18 µl, based on mouse weight) was paired with extinction of the drug availability light. Responses on the inactive lever were recorded but had no scheduled consequence. Each daily session lasted up to 6 h or until mice administered 30 mg/kg cocaine.

#### Dose response

Dose-response was tested over two consecutive days. Each day, mice had 30 min access to the acquisition dose (1 mg/kg) followed by access to descending cocaine doses, with 1 h/dose, on a FR1 (Day 1: 3.2, 1.5, 1.0, 0.75; Day 2: 0.5, 0.25, 0.125, 0.075 mg/kg/infusion).

#### Progressive ratio

Over four consecutive sessions, mice lever press to earn  different cocaine doses under a progressive responding ratio that increased according to the following equation  [26]:

$$presses\,required = \left[ {5e\left( {reward\,number\, \ast \,0.20} \right)} \right] - 5$$

Each session lasted up to 5 h or ended 1 h after the last reward if the next ratio was not reached. Breakpoint was defined as the number of presses made to earn the last cocaine infusion.

#### Cocaine seeking

Responding on the active and inactive levers was measured under extinction conditions (i.e., cocaine seeking) after 0, 14, and 120 days of forced cocaine abstinence. During abstinence, mice were not exposed to cocaine, or cocaine-related cues. The drug availability light was illuminated, but presses did not result in cocaine delivery. Sessions lasted up to 6 h, or until the mouse received the equivalent of 30 mg/kg cocaine.

### Electrophysiology

Mice were decapitated and the brain was placed in cutting solution. Sagittal slices (240 µm) were prepared (Leica VT-1000) and maintained at 31–33 ˚C in oxygenated aCSF. *Adora2aCre;Drd1-tdTomato* and iMSN-Drd2HET;*Drd1-tdTomato* mice expressing ChR2 in iMSNs received saline or cocaine (15 mg/kg) daily for 5 days. Two to five days after the last injection GABA-A receptor-mediated synaptic responses were recorded under whole-cell voltage clamp in tdTomato-positive MSNs in the NAc core. See Supplemental Methods for details. Optically-evoked inhibitory post-synaptic currents (oIPSCs) were triggered every 20 s by a single light pulse (473 nm; 0.2–1 ms duration) delivered through a fiber optic (200 µm/0.22 NA, ThorLabs) connected to a laser (25 mW, CrystaLaser). Synaptic blockers were used to isolate GABAergic responses (5 µM NBQX, 10 µM CPP, and 2 µM CGP). Quinpriole (1 µM) and sulpiride (1 µM) were bath applied for 5 or 10 min, respectively. For measurements of AMPA/NMDA ratios electrically-evoked excitatory post-synaptic currents (eEPSCs) were triggered using a monopolar glass electrode filled with aCSF placed ~100 µm away from the recording site in the NAc core. eEPSCs were recorded in the presence of the GABA-A receptor antagonist gabazine (5 µM) and d-serine (10 µM). The AMPA and NMDA components were measured at −70 mV and +40 mV, respectively, and the NMDA current was pharmacologically isolated with NBQX (10 µM). AMPA/NMDA ratio was calculated as the peak AMPA amplitude relative to the peak NMDA amplitude, and rise (ms, 10–90% of peak) and decay (ms, 10–90% of peak) times for AMPA and NMDA components were also determined. Data were acquired using Multiclamp 700B (Molecular Devices), filtered at 1 kHz, digitized at 5 kHz, and analyzed using pClamp (ClampFit, v.10.3).

### Statistics

Analyses were performed in Prism (GraphPad). Data from D1-like agonist-induced locomotion, cross-sensitization, cocaine self-administration, western blot, immunohistochemistry, and electrophysiology experiments were analyzed using 2-way ANOVA, with the addition of repeated measures as appropriate. Fiber density was analyzed by independent samples t-test. Significant main effects or interactions were followed-up with pairwise t-tests corrected for multiple comparisons. Results were considered significant at an alpha of 0.05. All data are presented as mean ± SEM.

## Results

### Cross-sensitization between cocaine and a D1-like receptor agonist suggests a common mechanism

**Fig. 1:**
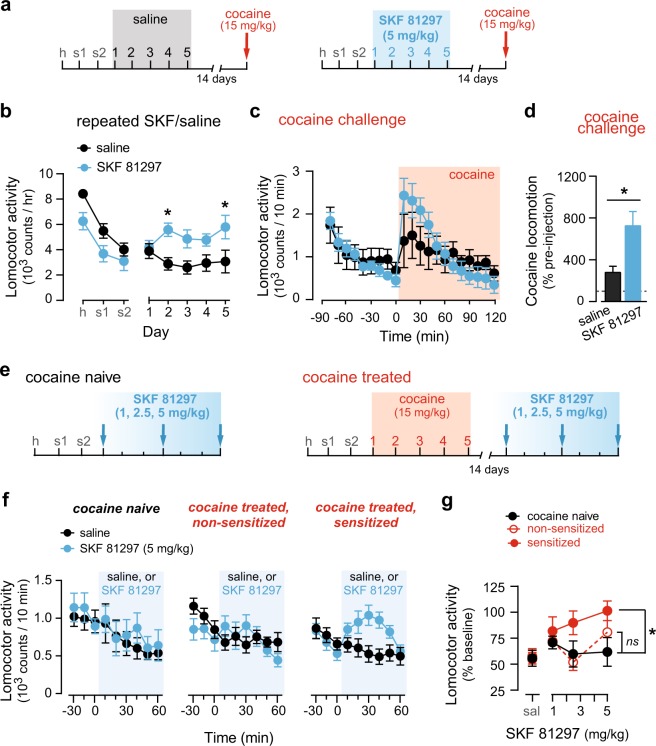
Cross-sensitization between cocaine and a D1-like agonist suggests a common mechanism. **a** Experimental timeline. Mice were treated with saline- (gray) or SKF-81297 (blue) for 5 days and all mice were challenged with a cocaine injection 14 days later. **b** Horizontal locomotor activity during habituation (h), two saline sessions (s1, s2) and during 5 daily sessions of treatment with saline (black) or SKF-81297 (blue). Posthoc t-test: Saline vs. SKF-81297: *p *<* 0.05. **c** Horizontal locomotor activity on cocaine challenge day before and after cocaine injection (red box) for mice pretreated with saline (black) or SKF-81297 (blue). **d** Normalized total counts during 1-h post cocaine challenge injection as a function of pre-challenge baseline for each mouse. Unpaired *t*-test, *p *<* 0.05. **e** Experimental timeline. Cocaine-naive (left) or cocaine-pretreated (right, red box) mice received escalating SKF-81297 doses (blue box). **f** Horizontal locomotor activity before and after saline (black) or SKF 81297 (blue, 5 mg/kg) administration for mice with no previous cocaine experience (cocaine naive, left plot) or mice pretreated with cocaine that did not show cocaine sensitization (middle plot) and those that did show cocaine locomotor sensitization (right plot, “sensitized”). **g** Locomotor activity to all doses of SKF-81297 tested for each group: cocaine naïve (black), cocaine-treated “non-sensitized” (red open), and cocaine-treated “sensitized” mice (red filled). Data is normalized to same-day, pre-injection baseline. Posthoc *t-*tests: * *p* *<* 0.05

We hypothesized that increased sensitivity of D1R signaling may underlie the heightened behavioral plasticity to repeated cocaine seen in mice lacking D2Rs in iMSNs (iMSN-Drd2KO). To asses this, we first tested cross-sensitization between a D1-like receptor agonist and cocaine in wild-type mice (Fig. 1a). Repeated SKF-81297 administration (5 mg/kg for 5 days) increased locomotion compared to saline-treated controls (Day × Drug: *F*_4,40_ = 3.30, *p* *<* 0.05, *n* = 6/group; Fig. 1b). Follow-up post-hoc comparisons revealed that acute SKF-81297 did not elicit a locomotor response above saline but did increase locomotion significantly from saline on Days 2 and 5 (*t*_50_ = 2.91–2.94, *p’s* *<* 0.05). On cocaine challenge day, baseline locomotion was similar between mice pre-treated with saline or SKF-81297, but cocaine-induced locomotion was higher in mice pre-treated with SKF-81297 (Time × Drug: *F*_20,180_ = 3.34, *p* *<* 0.0001, *t*_9_ = 3.18, *p* *<* 0.05; Fig. 1c, d).

We performed the converse experiment and tested whether cocaine sensitized mice showed a higher SKF-81297 locomotor response (Fig. 1e). A sensitized cocaine response was defined as a 15% increase in the locomotor response on day 5 compared to the acute response. Repeated cocaine treatment (15 mg/kg for 5 days) induced a sensitized response in 13 out of 21 (~60%) wild-type mice. Cocaine sensitized mice showed a leftward shift in the dose-response to SKF-81297 challenge compared to cocaine-naïve mice (Cocaine: *F*_1,61_ = 4.89, *p* *<* 0.05, *n* = 7-13/group; Fig. 1g). Thus, cocaine sensitization facilitated the locomotor response over saline at lower doses of SKF 81297 (Fig. 1f). Further, mice that were cocaine-treated, but did not sensitize, did not show a locomotor response to SKF 81297 and were not different from cocaine naïve mice (no main effect or interactions of cocaine and SKF).

### D2R deletion from iMSNs sensitizes the behavioral response to a D1-like agonist

**Fig. 2:**
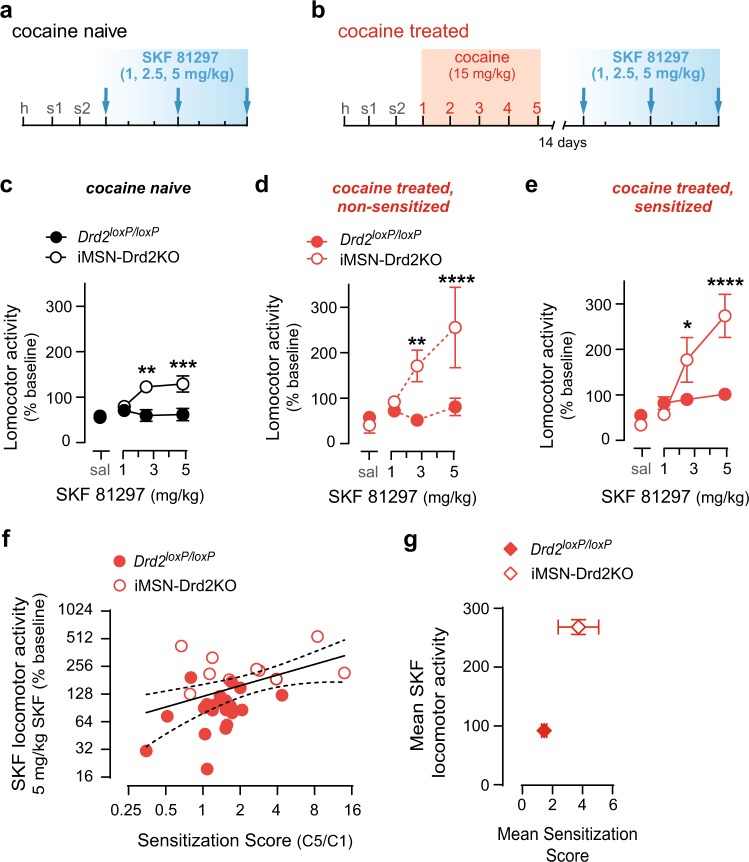
Targeted deletion of D2Rs from striatal medium spiny neurons induces behavioral hypersensitivity to D1Rs. **a**, **b** Experimental timeline. Cocaine-naive (**a**) or cocaine-pretreated (**b**) mice received escalating SKF-81297 doses (blue box). **c**–**e** Horizontal locomotor activity in response to SKF-81297 injection normalized to pre-injection baseline for cocaine-naive (**a**) and cocaine pretreated non-sensitized (**d**) and cocaine pretreated sensitized (**e**) *Drd2*^*loxP/loxP*^ (filled) and iMSN-Drd2KO (open). Posthoc t-tests: *Drd2*^*loxP/loxP*^ vs. iMSN-Drd2KO: *p *<* 0.05, **p *<* 0.01, ***p < 0.001, ****p < 0.0001. **f** Correlation between sensitization score (ratio of activity on cocaine day 5/cocaine day 1; C5/C1) and the acute locomotor response to SKF-81297 (5 mg/kg) for individual *Drd2*^*loxP/loxP*^ (filled) and iMSN-Drd2KO (open) mice (*R* *=* 0.42, *p* < 0.05). **g** Mean ± SEM of sensitization score and SKF-81297-induced locomotion (5 mg/kg) for *Drd2*^*loxP/loxP*^ (open) and iMSN-Drd2KO (filled)

We next assessed the sensitivity to a D1-like agonist in cocaine naïve mice lacking D2Rs selectively in iMSNs (iMSN-Drd2KO; Fig. 2a, b). We found they show a leftward shift in the dose-response to SKF-81297 compared to *Drd2*^*loxP/loxP*^ controls, as indicated by locomotor activation at lower doses of SKF 81297 (Dose × Genotype: *F*_3,39_ = 6.10, *p* *<* 0.01, *n* = 7–8/genotype; Fig. 2c). Cocaine naive iMSN-Drd2KO mice displayed a significant increase in locomotion above saline levels at 2.5 mg/kg and 5 mg/kg SKF-81297 (t_39_ = 4.8, *p* < 0.0001; t_39_ = 5.2, *p* < 0.0001), and when compared to *Drd2*^*loxP/loxP*^ littermate controls (t_52_ = 3.7, *p* < 0.01; t_52_ = 3.9, *p* < 0.001). Thus, selective deletion of the D2Rs from iMSNs results in behavioral hypersensitivity to a D1-like receptor agonist. Importantly, this effect did not seem to be due to the lower baseline locomotion in the iMSN-Drd2KO mice, as the enhanced locomotion to a D1-like agonist was reflected as a leftward shift in the dose-response, and not just an increase in the maximal response at the highest dose, wherein lower doses of SKF-81297 facilitated a locomotor response. This shift in the dose-response to SKF-81297 was also *Drd2* gene-dose-dependent, with iMSN-Drd2HET mice and iMSN-Drd2KO mice showing a locomotor response at lower SKF-81297 doses than littermate controls (Fig. 4e, S6).

### Enhanced cross-sensitization between a D1-like agonist and cocaine in iMSN-Drd2KO mice

Cross-sensitization between cocaine and a D1-like agonist was also enhanced in iMSN-Drd2KO mice (Fig. 2b). Seventy percent (7 out of 10) of iMSN-Drd2KO mice that received cocaine for 5 days met criteria for cocaine sensitization, which was comparable to the percentage of littermate controls that developed sensitization (Fishers Exact test: *p* *>* 0.99). Cocaine-sensitized iMSN-Drd2KO mice showed a leftward shift in the dose-response to SKF-81297 compared to cocaine-sensitized littermate *Drd2*^*loxP/loxP*^ mice, wherein lower doses of the D1-like agonist-induced a locomotor response in the iMSN-Drd2KOs (SKF × Genotype: *F*_3,58_ = 7.87, *p* *<* 0.001, *n* = 7-13/genotype; Fig. 2e). This was especially evident at the 2.5 mg/kg (*t*_58_ = 2.87, *p* *<* 0.05) and 5 mg/kg doses (*t*_58_ = 5.69, *p* *<* 0.0001). Interestingly, iMSN-Drd2KO mice that were cocaine-treated but did not develop cocaine sensitization also showed a shift in the SKF-81297 dose-response compared to the littermate controls (SKF × Genotype: *F*_3,31_ = 5.94, *p* *<* 0.01; Fig. 2d). In iMSN-Drd2KO mice, SKF-81297 induced locomotion above saline levels at 2.5 mg/kg and 5 mg/kg (*t*_31_ = 3.07, *p* *<* 0.05; *t*_31_ = 5.06, *p* *<* 0.0001). This contrasts with the non-sensitized littermate *Drd2*^*loxP/loxP*^ mice, which did not show a significant locomotor response at any SKF-81297 dose. Thus, iMSN-Drd2KOs show cross-sensitization between a D1-like agonist and cocaine similar to *Drd2*^*loxP/loxP*^ controls, but the extent of this effect is enhanced. Indeed, the magnitude of the acute SKF-81297 locomotor response was positively correlated with the magnitude of cocaine sensitization when both genotypes were analyzed together, with iMSN-Drd2KO mice showing greater responses on both parameters (*R*^2^ = 0.18, *p* *<* 0.05; Fig. 2f, g). Additionally, the D1R hypersensitivity observed in iMSN-Drd2KOs does not appear to be driven by a ceiling effect in *Drd2*^*loxP/loxP*^ controls for SKF-81297 locomotion because control mice are able to run more following repeated cocaine (Figure S1). Thus, cocaine sensitization induces behavioral hypersensitivity of the D1R, and this effect is recapitulated, and possibly facilitated, by targeted *Drd2* gene deletion from iMSNs.

### Enhanced D1R signaling in mice with low striatal D2Rs

**Fig. 3:**
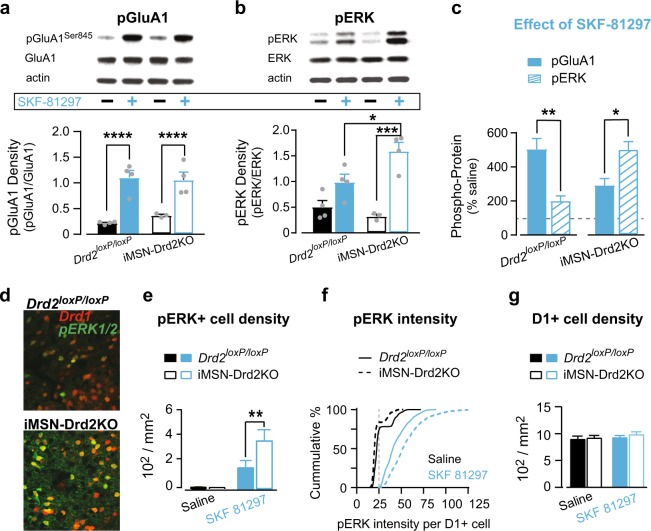
Shift in D1R signaling in direct-pathway MSNs from the NAc in mice lacking striatal D2Rs in iMSNs. **a**, **b** Western blot analysis from NAC tissue samples showing phosphorylated GluA1 at PKA-dependent serine 845 residue (pGluA1) and total protein (**a**) and phosphorylated ERK1/2 (pERK1/2) and total protein (**b**). Top, representative images of two samples of each treatment. Bottom, densitometry values for phospho-protein normalized to total protein after saline (black) or SKF-81297 (blue) in littermate *Drd2*^*loxP/loxP*^ (filled) and iMSN-Drd2KO mice (open). pGluA1: Main effect of Drug: ****p *<* 0.0001. pERK1/2: Posthoc *t*-tests, SKF-81297 vs. saline: ***p *<* 0.001; SKF-treated *Drd2*^*loxP/loxP*^ vs. iMSN-Drd2KO: *p *<* 0.05. **c** SKF-81297-induced phosphorylation of GluA1 (filled) and ERK1/2 (striped) in littermate *Drd2*^*loxP/loxP*^ (left) and iMSN-Drd2KO mice (right). Normalized levels of pGluA1 and pERK1/2 in NAc samples from SKF-81297 treated mice are expressed as a percentage of average levels in NAc samples of saline-treated mice. Unpaired *t*-tests: pGluA1 vs. pERK1/2: *p *<* 0.05; **p *<* 0.01. **d** Confocal images of *Drd1*-expressing neurons (red) and immunohistochemistry labeling for pERK (green) in NAc of control *Drd2*^*loxP/loxP*^ (top) and iMSN-Drd2KO (bottom) following SKF-81297 (5 mg/kg) injection. **e** Density of pERK-positive D1R-containing neurons in *Drd2*^*loxP/loxP*^ (filled) and iMSN-Drd2KO (open) mice after saline (black) and SKF-81297 (blue) administration. Posthoc *t*-test: SKF-treated *Drd2*^*loxP/loxP*^ vs. iMSN-Drd2KO: **p *<* 0.01. **f** Cumulative histogram showing the frequency distribution of pERK immunostaining intensity in D1R-containing neurons after saline (black) and SKF-81297 (blue) for control *Drd2*^*loxP/loxP*^ (solid) and iMSN-Drd2KO mice (dashed) relative to threshold (gray dashed line). **g** Density of D1R-containing neurons in the NAc of *Drd2*^*loxP/loxP*^ and iMSN-Drd2KO after saline or SKF-81297

We previously reported that mRNA expression levels for the *Drd1* gene are not changed in iMSN-Drd2KO [20]. Thus, we hypothesized that the behavioral hypersensitivity to a D1-like agonist observed in these mice was due to an upregulation in D1R-mediated intracellular signaling. To test this, we measured protein kinase A (PKA)-dependent phosphorylation of GluA1 (pGluA1) and MEK-dependent phosphorylation of ERK1/2 (pERK1/2) in the NAc and DMS following D1R activation using Western blot. Total protein levels of GluA1, ERK1/2, and actin did not differ as a function of drug treatment or genotype in either region (Figure S2–S3). In the NAc, acute SKF-81297 (5 mg/kg) administration increased pGluA1 levels similarly for iMSN-Drd2KO and *Drd2*^*loxP/loxP*^ mice compared to saline-treatment (Drug: *F*_1,11_ = 48.3, *p* *<* 0.0001, *n* = 3–4/group; Fig. 3a). In contrast, acute SKF-81297 increased levels of pERK1/2 disproportionately in the NAc of iMSN-Drd2KO mice compared to littermate controls (Drug × Genotype: *F*_1,11_ = 7.54, *p* *<* 0.05; Fig. 3b). Phosphorylated levels GluA1 and ERK1/2 following SKF-81297 were normalized to their saline-treated levels (Fig. 3c). This analysis revealed that a shift from PKA- to MAPK-mediated signaling following D1R activation in the NAc of mice lacking D2Rs in iMSNs. While SKF-81297 enhanced pGluA1 in both genotypes, the levels of pERK1/2 were significantly greater in iMSN-Drd2KO mice (*t*_6_ = 3.07, *p* *<* 0.05). This effect was reversed in littermate controls, which showed greater D1R-induced pGluA1 levels relative to pERK levels (*t*_6_ = 4.3, *p* *<* 0.01).

In the DMS, SKF-81287 treatment only increased pGluA1 levels and did so equally between genotypes (Drug: *F*_1,11_ = 69.4, *p* *<* 0.0001, Figure S3). No changes in pERK1/2 levels were observed following SKF-81297 in the DMS of either genotype. Further, the normalization analysis showed that SKF-81297 selectively facilitates pGluA1 over pERK1/2 signaling in the DMS.

To determine the cell-type specificity of the D1R-mediated signaling, we performed immunohistochemistry for pERK1/2 in the NAc of mice expressing a red fluorescent protein in D1R-expressing MSNs (Drd1-tdTomato mice). Following acute saline, the density of pERK1/2 positive D1R-expressing dMSNs was low in both genotypes. Acute SKF-81297 (5 mg/kg) increased the density of pERK1/2 positive dMSNs in both genotypes (Genotype × Drug: *F*_1,65_ = 4.2, *p* *<* 0.05, *n* = 15-18 cells/genotype; Fig. 3d, e). However, this D1R-dependent enhancement was significantly greater in iMSN-Drd2KO than *Drd2*^*loxP/loxP*^ controls (*t*_65_ = 2.94, *p* *<* 0.01; Fig. 3e, f). Additionally, the density of D1R-expressing neurons in the striatum was similar between genotypes, suggesting that higher density of *Drd1* expressing neurons is not contributing to the D1R hypersensitivity in iMSN-Drd2KOs (Fig. 3g). These data indicate that loss of D2Rs in iMSNs is accompanied by an upregulation of D1R-mediated intracellular signaling, specifically within striatal D1R-containing cells.

It has been proposed that ERK signaling in striatal D1R-containing MSNs functions as a coincidence detector between and D1R-mediated dopamine signaling and glutamate transmission to promote cocaine locomotor sensitization [27–29]. Given that iMSN-Drd2KO mice show a shift from PKA- to MAPK-mediated signaling in NAc dMSNs, we hypothesized this could lead to changes in striatal glutamate transmission and predicted that iMSN-Drd2KO mice would be more sensitive to cocaine-mediate potentiation of glutamate transmission. We performed whole-cell voltage-clamp recordings to measure glutamatergic synaptic responses from dMSNs in the NAc of iMSN-Drd2KO and littermate controls and tested the effect of a single cocaine injection which normally is not sufficient to potentiate excitatory inputs. Under these conditions we found no difference between genotypes in the AMPA/NMDA ratio at baseline, nor was there an effect of a single cocaine injection on the AMPA/NMDA ratio (Figure S5). Additionally, Pearson correlational analyses found no significant relationship between the AMPA/NMDA ratio with the days since last saline or cocaine injection for either genotype (*p’s* *=* 0.82–0.55). The rise-time and decay-time kinetics of the AMPA and NMDA receptor currents were also similar between genotypes, with only the iMSN-Drd2KO showing a trend for slightly faster decay of the NMDA receptor current (Unpaired *t*-test: *t*_13_ = 1.96, *p* = 0.07; Figure S5 c,d). Furthermore, cocaine did not alter these current kinetics (2-way ANOVA; No main effects of Drug or Genotype or Drug × Genotype interaction, *p’s* *=* 0.22–0.96; Figure S5).

Additionally, the Gi-coupled dopamine D3 receptor (D3R) is hypothesized to restrain striatal D1R activity and gate behavioral sensitization [30, 31]. We thus hypothesized that striatal *Drd3* mRNA expression would be downregulated in iMSN-Drd2KO and tested this by performing quantitative PCR in striatal tissue from naïve iMSN-Drd2KO mice and littermate controls. We found no evidence of altered striatal *Drd3* mRNA expression (Figure S4).

### Heterozygous deletion of striatal D2Rs as a model with improved face validity

**Fig. 4:**
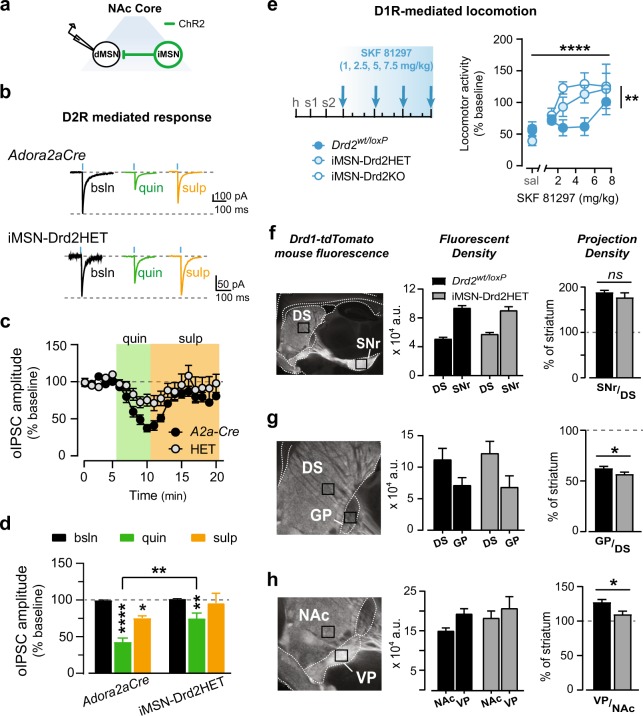
Partial reduction of striatal D2Rs is sufficient to trigger D1R hypersensitivity and structural alterations to striatopallidal neurons. **a** Schematic of the experimental approach showing ChR2 expression in iMSNs and whole-cell recording in neighboring dMSNs in NAc core. **b** Representative traces of optogenetic-evoked inhibitory post-synaptic currents (oIPSC) recorded at baseline (bsln), in quinpirole (1 µM), and sulpiride (1 µM) in slices from *Adora2aCre* (top) or iMSN-Drd2HET (bottom) mice. **c** Time course of oIPSC amplitude normalized to baseline before quinpirole in *Adora2aCre* (black) or iMSN-Drd2HET (gray). **d** Summary of the quinpirole and sulpiride effects in control *Adora2aCre* and iMSN-Drd2HET mice. Posthoc *t*-tests: Baseline vs. Drug: *p *<* 0.05, **p *<* 0.01, ****p < 0.0001; *Drd2*^*loxP/wt*^ vs. iMSN-Drd2HET at quinpirole: **p *<* 0.01. **e** Left, Timeline of behavioral experiment (h, habituation, s, saline). Right, Horizontal locomotor activity in response to escalating doses of SKF-81297, shown as a percent of same-day pre-injection baseline, in cocaine-naïve *Drd2*^*loxP/wt*^ control (dark blue), iMSN-Drd2HET (light blue), and iMSN-Drd2KO mice (white). 2-way ANOVA: Main effect of Genotype: **p < 0.01; 2-way ANOVA: Main effect of SKF-81297: ****p *<* 0.0001. **f**–**h** Left, representative images of parasagittal brain sections showing red-fluorescence from D1R-containing neurons in *Drd1*-tdTomato mice and overlay of the region used for quantification in the striatum (DS or NAC) and the projection areas (SNr, GP, VP). Middle plots represent the mean ± SEM of the fluorescence intensity (in arbitrary units, a.u.) in the striatal and projection regions for *Drd2*^*loxP/wt*^ (black) and iMSN-Drd2HET mice (gray). Right plots represent the mean ± SEM of the normalized fluorescence intensity of the projection regions as a function of the striatal region for *Drd2*^*loxP/wt*^ (black) and iMSN-Drd2HET mice (gray). **f** Unpaired *t*-test: ns = not significant. **g**, **h** Unpaired *t*-tests: *Drd2*^*loxP/wt*^ vs. iMSN-Drd2HET: *p < 0.05

iMSN-Drd2KO mice have two floxed *Drd2* alleles (homozygotes) and show a pronounced reduction in *Drd2* mRNA (~80%; [12]). However, the clinical literature suggests that even a small reduction of ~10–16% in striatal D2R availability is associated with stimulant abuse [6, 32]. We were interested in testing a mouse model with partial reduction of D2Rs from iMSNs and improved face validity, and therefore used iMSN-Drd2HET mice, which have an ~40% reduction in *Drd2* mRNA ([20], Figure S6). To determine the extent of functional loss of D2Rs in iMSNs in iMSN-Drd2HET mice, we tested the ability of the D2-like agonist quinpirole to inhibit GABA transmission from iMSN axon collaterals (Fig. 4a). In control *Adora2a-Cre* mice, quinpirole (1 µM) inhibited optogenetic-evoked inhibitory post-synaptic current (oIPSC) amplitude by 57 ± 5 % (*n* = 12 cells; Fig. 4b-d). In iMSN-Drd2HET mice, quinpirole was significantly less effective (26 ± 7 % inhibition; Genotype × Drug: *F*_2,55_ = 3.60, *p* *<* 0.05; t_55_ = 3.84, *p* *<* 0.001). Additionally, we tested whether repeated cocaine could further downregulate D2Rs in iMSN-Drd2HET mice. We found no evidence of D2R downregulation in either genotype (see Supplemental Results; Figure S7). Thus, compared to iMSN-Drd2KO mice which show no quinpirole-mediated inhibition [12], iMSN-Drd2HET mice have an intermediate, functional loss of D2Rs in iMSNs.

### D1R hypersensitivity is observed following single allele deletion of *Drd2* in iMSNs

The D1R-mediated behavioral response was also enhanced in iMSN-Drd2HET mice (Fig. 4e). iMSN-Drd2HET and iMSN-Drd2KO mice both showed enhanced locomotor activation to a SKF-81297 dose-response compared to *Drd2*^*loxP/loxP*^ littermates (Genotype: *F*_2,93_ = 6.8, *p* *<* 0.01; *n* = 7–9/genotype). Interestingly, SKF-81297 dose-dependent increased locomotion was similar in iMSN-Drd2HET and iMSN-Drd2KO mice (*t*_93_ = 2.0, *p* *=* 0.09), suggesting that the partial reduction in striatal D2R levels is sufficient to trigger the D1R behavioral hypersensitivity.

### Structural plasticity at D1R-expressing MSN projections

We found evidence of structural alterations within the striatopallidal circuit when measuring fluorescence intensity in the projection areas of dMSNs (globus pallidus, GP; ventral pallidum, VP; substantia nigra, SNr) relative to the DS or NAc (^SNr^∕_DS,_^GP^∕_DS,_ and ^VP^∕_NAc_; Fig. 4f-h). In iMSN-Drd2HET mice expressing td-Tomato in D1R-containing neurons, the normalized fluorescent projection density was lower in the GP and VP (relative to the DS and NAc, respectively) compared to littermate controls (^GP^∕_DS_: *t*_72_ = 2.0, *p* *<* 0.05, *n* = 33–41 sections/genotype; ^VP^∕_NAc_: t_65_ = 2.16, *p* *<* 0.05, *n* = 19–48 sections/genotype; Fig. 4g, h). There was no difference between genotypes in the relative fluorescence of projections to the SNr (Fig. 4f). Analysis of the raw fluorescent density revealed that decreased projection density to pallidal regions observed in iMSN-Drd2HET mice appears to be driven by a higher fluorescent density in the DS and NAc of the iMSN-Drd2HETs (Fig. 4f–h, middle panels). While analysis of the normalized projection density suggests lower density of bridging collaterals from dMSNs in mice with low striatal D2Rs, the raw fluorescent density data indicates that there may also be differences in intrastriatal collateral density. Further analysis using finer techniques to more accurately trace axons and map the density of intrastriatal and bridging axon collaterals is needed to further probe how the striatopallidal circuit is reorganized following targeted deletion of D2Rs from iMSNs.

### Cocaine seeking and taking in iMSN-Drd2HET mice

**Fig. 5:**
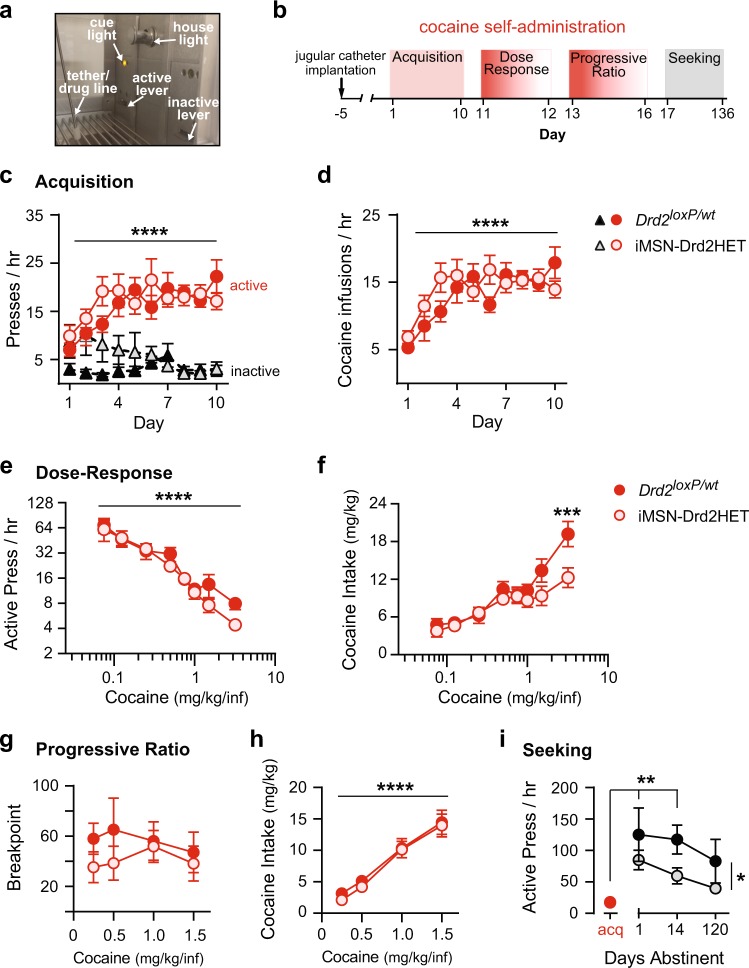
Reduction in striatal D2Rs does not enhance intravenous cocaine self-administration in mice. **a** Operant chamber for intravenous cocaine self-administration experiment. **b** Experimental timeline. **c**, **d** Rate of responding on active (red) and inactive (black) lever (**c**) and rate of earned cocaine infusions (**d**) over 10 days of acquisition of cocaine self-administration (FR1, 1 mg/kg/infusion, ≤ 30 mg/kg cocaine or 6 h/day) for *Drd2*^*loxP/wt*^ (dark) and iMSN-Drd2HET (light) mice. 2-way ANOVA: Main effect of Day: ****p *<* 0.0001. **e, f** Rate of active lever responding (**e**) and total cocaine intake (**f**) as a function of the cocaine unit dose delivered during two dose-response sessions (FR1, escalating unit-dose every hour) for *Drd2*^*loxP/wt*^ (dark) and iMSN-Drd2HET (light) mice. 2-way ANOVA: Main effect of Cocaine Dose: ****p *<* 0.0001; Posthoc *t*-test: *Drd2*^*loxP/wt*^ vs. iMSN-Drd2HET. **g,** **h** Breakpoint values (number of lever presses emitted to receive the last cocaine infusion) reached (**g**) and total cocaine intake (**h**) during sessions with a progressive ratio schedule of reinforcement as a function of the cocaine unit dose delivered for *Drd2*^*loxP/wt*^ (dark) and iMSN-Drd2HET (light) mice. 2-way ANOVA: Main effect of Cocaine Dose: ****p *<* 0.0001. **i** Cocaine seeking under extinction conditions was measured as rate of active lever responding over increasing duration of cocaine abstinence for *Drd2*^*loxP/wt*^ (dark) and iMSN-Drd2HET (light) mice and compared to mean rate over the last 4 days of acquisition (red). 2-way ANOVA: Main effect of Genotype: *p *<* 0.05; Posthoc *t*-tests: Acquisition vs. Day 1 for both genotypes: ****p *<* 0.0001; Acquisition vs. Day 14 for both genotypes: **p *<* 0.01

Male and female iMSN-Drd2HET mice and *Drd2*^*loxP/wt*^ littermate controls were tested on operant intravenous cocaine self-administration across a range of procedures, including acquisition, dose-response, progressive ratio, and seeking (Fig. 5a, b). No differences were noted between males and females (Figure S8), thus data were collapsed across sex for greater powered analysis. Additionally, we analyzed the inactive lever responding across all self-administration procedures and found no consistent differences between genotypes in most stages tested, except for the first week of acquisition and at low doses during the dose-response (see Supplemental Results for details).

iMSN-Drd2HET mice showed a similar rate of active lever pressing over acquisition for cocaine (1 mg/kg/infusion) compared to *Drd2*^*loxP/wt*^ controls over 10 days (Day: *F*_9,299_ = 4.23, *p* < 0.0001, *n* = 16–17/genotype; Fig. 5c). Both genotypes increased active lever responding over the 10 days (no effect of genotype or genotype × day interaction). Additionally, the average rate of cocaine self-administration was similar between genotypes over the last 4 days of training (*Drd2*^*loxP/wt*^: 15.9 ± 1.2, iMSN-Drd2HET: 14.9 ± 1.0 rewards/h; *t*_31_ = 0.64, *p* *=* 0.53; Fig. 5d). We next tested whether iMSN-Drd2HET mice have enhanced sensitivity for cocaine reinforcement under a dose-response. Active lever responding decreased as the cocaine dose increased for both genotypes (Dose: *F*_7,191_ = 19.3, *p* *<* 0.0001; *n* = 14–15/genotype; Fig. 5e). Total cocaine intake per session increased as a function of the unit dose (Dose: *F*_7,191_ = 16.5, *p* *<* 0.0001); however, iMSN-Drd2HET mice administered less cocaine at the highest unit dose compared to littermate controls (Dose × Genotype: *F*_7,191_ = 1.82, *p* *=* 0.08; Genotype: *F*_7,191_ = 8.92, *p* *<* 0.01; post-hoc t-test *p* *<* 0.01; Fig. 5e).

Motivation to take cocaine was assessed using progressive ratio, wherein the operant requirement to earn each successive cocaine infusion increases exponentially. Total cocaine intake increased as a function of the unit dose (Dose: *F*_3,95_ = 37.2, *p* *<* 0.0001; Fig. 5h). However, there were no differences between genotypes in the breakpoint or total cocaine intake, suggesting iMSN-Drd2HET mice do not have altered motivation to take cocaine (Fig. 5g).

Lastly, we tested cocaine seeking under extinction conditions over increasing duration of drug abstinence. Abstinence has been shown to lead to greater drug craving and seeking behavior in humans and rodents, a phenomenon known as the incubation of craving [33, 34]. Both genotypes showed increased active lever responding on the first day of cocaine abstinence relative to acquisition (Abstinence: *F*_3,86_ = 7.9, *p* *<* 0.0001; *n* = 12–15/genotype; Fig. 5i). However, iMSN-Drd2HET mice exhibited decreased active lever pressing across all time-points (Genotype: *F*_1,86_ = 5.8, *p* *<* 0.05).

## Discussion

In this study, we used a targeted genetic deletion strategy to determine the consequence of downregulating D2Rs selectively from striatal iMSNs on the striatal circuitry and tested whether these alterations are sufficient to facilitate cocaine self-administration and locomotor sensitization. We found that selective downregulation of D2Rs in iMSNs triggers profound neuroadaptations in basal ganglia circuitry and function, including a shift toward MAPK-signaling in the NAc following D1R activation, behavioral hypersensitivity to a D1-like receptor agonist, and a structural reorganization of striatopallidal projections. This D1R hypersensitivity is associated with locomotor sensitization to repeated cocaine administration. Despite these alterations, however, reduction of D2Rs from iMSNs did not change cocaine self-administration under the conditions tested here. While it is still unclear how low D2Rs on iMSNs trigger the enhanced D1R signaling in dMSNs, we propose that the mechanism involves the known local synaptic connections between these two subclasses of MSNs [12, 39]. We speculate that balance of D2R and D1R activity in the striatum is tightly regulated and sensed by the strength of the local lateral inhibition between MSNs (more on this below).

### D1R hypersensitivity as a mechanism for cocaine locomotor sensitization

We report that iMSN-Drd2KO mice show a shift in D1R-mediated signaling from CREB-dependent PKA-singling to an ERK1/2 MAPKinase signaling pathway selectively in the NAc. These findings, while novel for this selective D2R deletion mouse model, are consistent with seminal findings from dopamine depletion models [49, 50]. Interestingly, the dopamine depletion mouse model shares Parkinsonian-like phenotypic similarities to our iMSN-Drd2KO mice, which also develop enhanced D1R-mediated activation of the ERK1/2 MAPKinase signaling pathway in direct pathway MSNs that is restricted to the NAc. Additionally, acute administration of a D1-like receptor agonist increased pGluA1 levels at serine-845 within the DMS, similar to previous reports using D1-like receptor agonists or cocaine [35–37]. While the cell-specificity of the pGluA1 increase cannot be determined with western blot analysis, a previous report showed that an adenosine A1 receptor agonist blocks D1R-mediated increases in pGluA1, suggesting the increase phosphorylation occurs in dMSNs [35].

Further, using immunohistochemistry we observed a D1R-mediated enhancement in pERK1/2 levels specifically in D1R-containing dMSNs, and show that this effect was greater in iMSN-Drd2KO mice. ERK has been proposed to act as a coincidence detector for glutamate- and dopamine-mediated signaling in the striatum [28], and is required for cocaine-induced potentiation of AMPA-mediated glutamate transmission onto dMSNs [27, 29]. However, despite this shift from primarily PKA to MAPK signaling, we observed no changes in glutamate transmission in iMSN-Drd2KO mice, as measured by AMPA/NMDA ratio. Together, these data provide evidence that low levels of D2Rs in iMSNs triggers specific alterations to D1R-mediated signaling in dMSNs that are striatal region dependent, which we speculate underlies the D1R hypersensitivity that facilitates cocaine sensitization.

### Interactions between direct and indirect-pathway neurons and the balance of D2R and D1R activation

This study provides clear evidence of a tight regulatory balance between D2R and D1R activity and signaling, despite the cellular segregation. How can this crosstalk take place? We propose that D1R-expressing and D2R-expressing MSNs influence each other via the extensive web of local axon collaterals [38, 39]. In this way, D2Rs expressed in iMSNs are poised to influence the activity of neighboring dMSNs by limiting the lateral inhibition from iMSNs and disinhibiting action potential firing of dMSNs [12]. Consequently, reduction of D2R levels in iMSNs increases lateral inhibition onto neighboring dMSNs, which we previously observed as higher tonic GABA transmission on dMSNs, less in vivo firing and a motor impairment [20]. We speculate that loss of D2Rs and subsequent heightened GABA tone is sensed by D1R-expressing dMSNs thus triggering an upregulation of D1R-mediated signaling as an adaptive response to facilitate dMSN activity and promote motor output.

The expression levels of D2Rs in the striatum also exerts bi-directional control over the extent of bridging collaterals from D1R-containing dMSNs that project to the pallidum. Overexpression of D2Rs in iMSNs increases the density of bridging collaterals in the pallidum and full Drd2KO mice showed lower density of these projections [40]. Antagonist for D2Rs also caused a reduction in projection density and this effect seems to be a consequence of changes in the excitability of iMSNs [40]. Consistent with these published findings, here we showed that selective reduction D2Rs from iMSNs is sufficient to decrease the relative density of bridging collaterals to the globus and ventral pallidum (GP and VP). The functional relevance of the bridging collaterals is still unclear, and as such it is hard to speculate on the consequences of these structural changes. It seems safe, though, to think that the structural changes are either a direct consequence of the changes in iMSNs and their synaptic inputs to the GP and VP, or an adaptive response to them. Either way, we consider that these findings provide clear evidence of long-lasting structural changes to the D1R-containing direct-pathway neurons upon downregulation of D2Rs in the indirect-pathway neurons. Thus, similar to chronic cocaine exposure, downregulation of D2Rs in iMSNs seems to alter striatal function and connectivity to induce a “pre-sensitized” or “primed” state.

The observed D1R hypersensitivity is a potential mechanism explaining why locomotor sensitization is preserved and facilitated in iMSN-Drd2KOs, despite the blunted acute cocaine locomotor response. We speculate that the D1R hypersensitivity in dMSNs could in principle be an adaptive response to the loss of D2Rs in iMSNs to overcome the bradykinesia and increased latency to move observed in animals with low D2Rs. These adaptive changes could then generate vulnerable circuitry for abuse by facilitating the development of D1R-dependent cocaine behaviors such as locomotor sensitization and conditioned place preference (CPP).

### D2Rs in cocaine self-administration: a complex role

Several factors influence how low striatal D2R activity contributes to cocaine abuse, including the cocaine dose, the cocaine behavior measured, and the degree and localization of D2Rs being downregulated [24, 41–45]. D2Rs expressed in dopamine neurons (i.e., “pre-synaptic”) and D2Rs expressed in striatal MSNs (i.e., “post-synaptic”) regulate different aspects of cocaine reinforcement, which is likely due to their distinct actions on synaptic transmission within the striatum [12, 20, 24, 46, 48]. Pre-synaptic D2Rs inhibit dopamine release, thus reduction of these D2 autoreceptors enhances dopamine transmission, locomotion, and incentive salience to cocaine-predictive cues. Post-synaptic D2Rs in iMSNs inhibit collateral GABA transmission between MSNs, which we showed can exert potent lateral inhibition within the striatum. Selective deletion of D2Rs in iMSNs causes bradykinesia and dampens the acute response to cocaine while facilitating the development of select responses to repeated cocaine administration, such as cocaine sensitization and CPP.

In the current cocaine self-administration experiments, mice with heterozygous deletion of D2Rs (iMSN-Drd2HET) were tested because they show less motor impairment than homozygous D2R deletion (iMSN-Drd2KO), reducing the potential of a motor confound. Using mice with heterozygous deletion generates an intermediate D2R reduction, which also has greater face validity. It is important to note that we limited daily cocaine intake to 30 mg/kg during acquisition to equalize cocaine history between genotypes and eliminate this as a possible confound. Under these conditions, iMSN-Drd2HET mice acquired cocaine self-administration at similar rates as littermate controls and there was no significant difference on intake between genotypes. It is possible that under other experimental conditions, such as providing unrestricted access to cocaine, a role for low D2Rs in iMSNs in enhancing cocaine taking could be revealed. The motivation to take cocaine was assessed by responding on a progressive ratio across multiple cocaine doses, and again no significant changes were identified. There was a trend for decreased breakpoint at lower cocaine doses, which would complement a previous report showing increased motivation for food reward in animals with D2R overexpression in iMSNs [47]. Surprisingly, iMSN-Drd2HET mice also consumed less cocaine at the highest unit dose and showed lower seeking under extinction conditions. It is possible that the D1R hypersensitivity contributes to lowering the threshold for dMSN activation in response to high-dose cocaine, and thus may explain why iMSN-Drd2HET mice self-administer less cocaine at high doses. In other words, iMSN-Drd2HET mice might be getting “more bang for their buck”. Also, it is likely that iMSN long-range projections to the VP are playing an important role in lowering the motivation for cocaine, as proposed in the previously published study where the motivation for food reward increased following D2R overexpression in iMSNs [47].

An alternative interpretation is that the lower cocaine intake at high cocaine doses and lower seeking during progressive abstinence are the result of general motor suppression in iMSN-Drd2HET mice. However, further analysis of rate of inactive lever responding showed no consistent decreases in iMSN-Drd2HET mice, suggesting the suppression was selective for the active lever. On the other hand, this analysis revealed possible impairment in the discrimination between active and inactive levers during the first week of acquisition, suggesting some learning impairments in iMSN-Drd2HET mice.

## Concluding remarks

Pre-existing low levels of striatal D2Rs triggers a shift in D1R-mediated signaling in dMSNs towards MAPK-signaling, a D1R behavioral hypersensitivity and reorganization of striatopallidal connectivity to facilitate behavioral plasticity to repeated cocaine administration. We speculate that imbalance in D2R-D1R activity is sensed by the enhanced lateral inhibition onto dMSNs, which triggers, by a mechanism yet to be characterized, a shift in D1R-mediated signaling to facilitate dMSN activity and motor output in an otherwise suppressed state. However, when striatal dopamine levels are high, such as in the presence of cocaine, hypersensitive D1Rs facilitate locomotion beyond the level of control mice with intact D2Rs and might predispose to an abuse vulnerable phenotype.

Together with our previously published findings examining the role of D2 autoreceptor[24, 46], these data reveal distinct roles for cell-type specific striatal D2Rs in regulating cocaine seeking and taking. The findings suggest the interesting possibility that the effects of low D2R levels in iMSNs and dopamine neurons might be additive, or even synergistic, with regards to generating an addiction vulnerable phenotype.

## Funding and Disclosure

This study was funded by the Center on Compulsive Behaviors, NIH via NIH Director’s Challenge Award program and the DDIR Innovation Award program to LKD and VAA, by an AMGEN fellowship (NIH-OD) to LE, by a Postdoctoral Research Associate (PRAT) fellowship from NIGMS (1Fi2GM117604-01) to MEB, by the Intramural Research Programs of the NIAAA and NINDS (ZIA-AA000421) to VAA, and by the U01 collaborative grant from NIAAA (U01AA023489) to DR and VAA. The authors declare no competing interests.

## Supplementary information

Figure S1-S8

Supplementary material
