# Supplementary material for: D1 receptor hypersensitivity in mice with low striatal D2 receptors facilitates select cocaine behaviors
Source: Neuropsychopharmacology. 2018 Dec 1;44(4):805–16. doi: 10.1038/s41386-018-0286-3 (PMC6372593; doi:10.1038/s41386-018-0286-3)
Supplement: Supplementary file 1 — Figure S1-S8 [file 41386_2018_286_MOESM1_ESM.pdf]

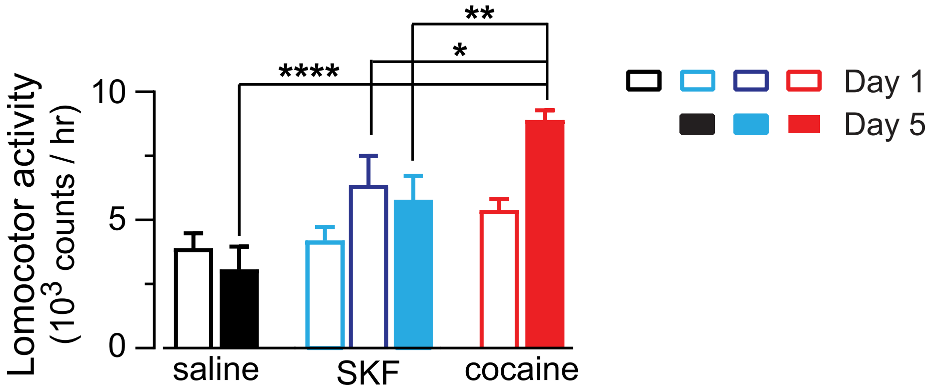

Figure S1. The D1-like agonist SKF 81297 at 5 mg/kg does not induce a ceiling effect in locomotion for wild-type mice. Horizontal locomotor activity is shown following saline (black), 5 mg/kg SKF 81297 (light blue), 7.5 mg/kg SKF-81297 (dark blue), and cocaine (15 mg/kg, red) treatment in *Drd2*$^{loxP/loxP}$ mice. Locomotor activity is shown after acute treatment (open bars) and after 5 consecutive days of treatment (filled bars) for each drug. 1-way ANOVA showed significant differences in locomotion after these different drug treatments ($F_{6,47} = 11.9$, $p < 0.0001$). Posthoc t-test showed that mice ran more following on Day 5 of cocaine versus Day 1 of 7.5 mg/kg SKF-81297 ($t_{47} = 2.70$, $p < 0.05$), Day 5 of 5 mg/kg SKF-81297 ($t_{47} = 3.68$, $p < 0.01$), and Day 5 of saline ($t_{47} = 6.90$, $p < 0.0001$).

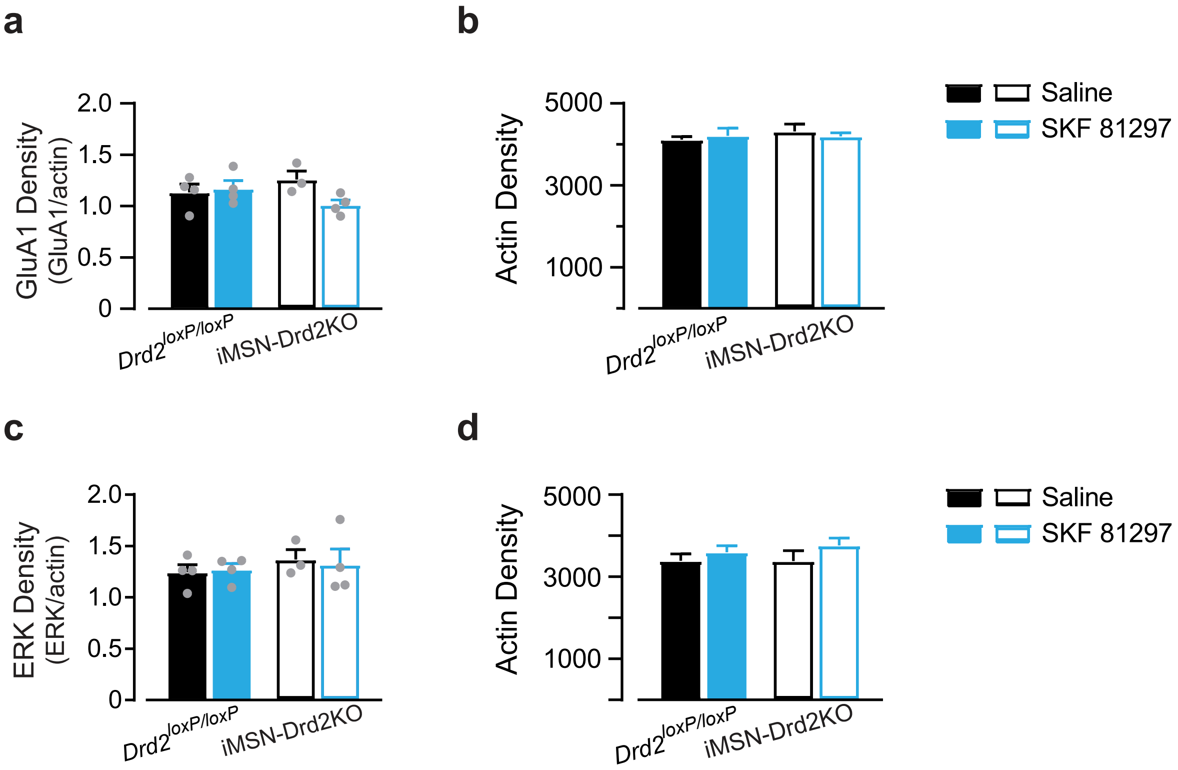

Figure S2. Levels of total GluA1, ERK, and actin are unchanged in the nucleus accumbens following acute treatment with a D1-like agonist. *Drd2*$^{loxP/loxP}$ and iMSN-Drd2KO mice were treated acutely with saline (black) or SKF 81297 (blue, 5 mg/kg) and Western blot analysis was performed for GluA1 and actin (a, b) and ERK1/2 and actin (c, d) in the nucleus accumbens. Densitometry values are shown for the total protein levels that correspond to the phosphorylated protein levels shown in Figure 3.

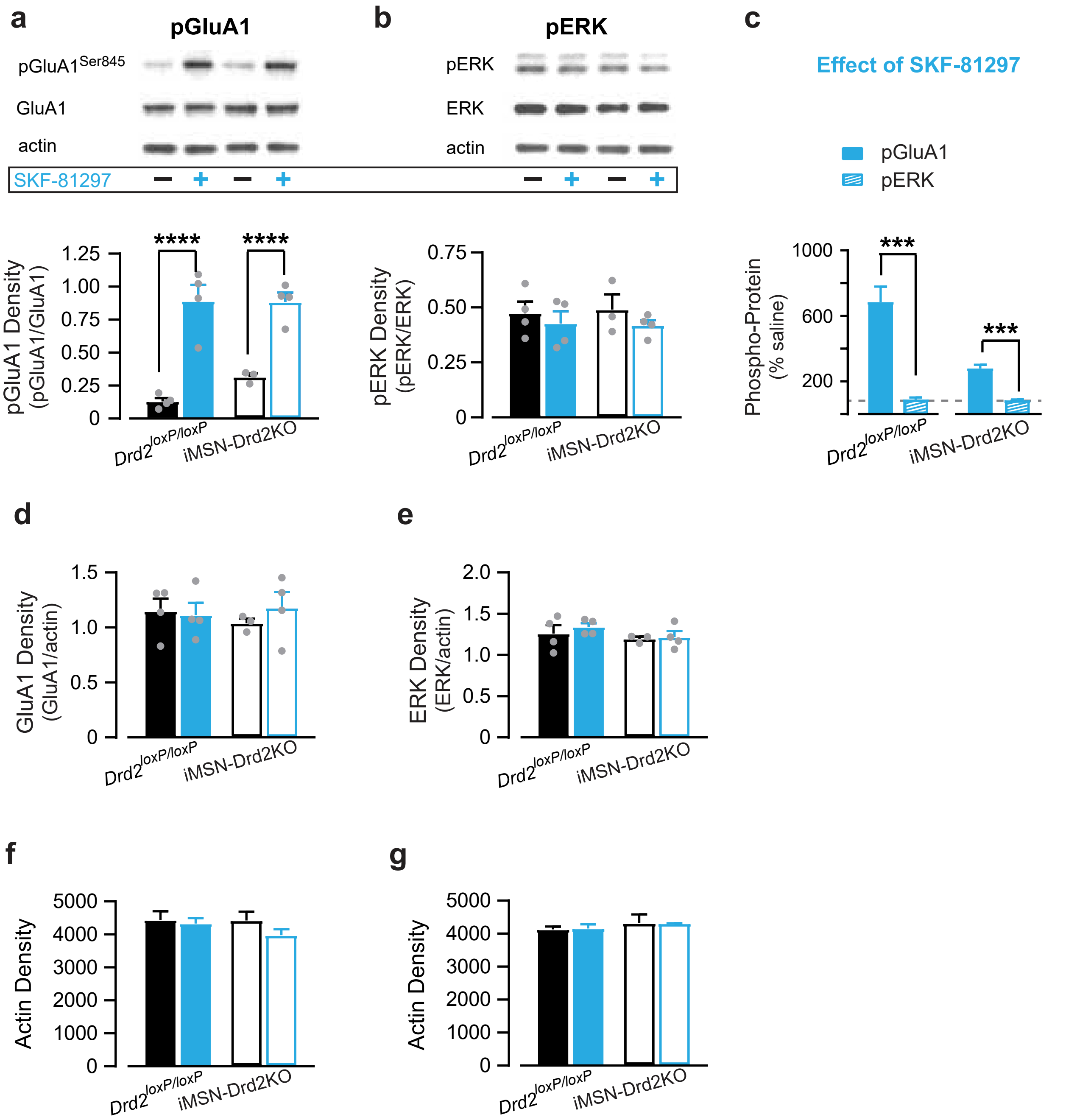

Figure S3. D1R-mediated increase in pGluA1 levels in the dorsal medial striatum is similar for iMSN-Drd2KO mice and littermate controls. (a, b) Western blot analysis of phosphorylated GluA1 at PKA-dependent serine 845 residue (pGluA1, a) and phosphorylated ERK1/2 (b) is shown for the dorsal medial striatum. Densitometry values for phosphorylated protein levels are normalized to total protein levels after SKF-81297 (blue) or saline (black) in *Drd2*$^{loxP/loxP}$ (filled) and iMSN-Drd2KO mice (open). Representative western blot bands are shown above their corresponding bar graph. (c) Levels of pGluA1 (blue filled) and pERK (blue striped) following acute SKF 81297 are presented as the average ratio to total GluA1 or ERK1/2, respectively, and expressed as a percentage of saline levels for *Drd2*$^{loxP/loxP}$ (left) and iMSN-Drd2KO mice (right). (e-g) Total protein levels for GluA1 (d) and ERK1/2 (e) and the corresponding actin levels (f-g) are shown below their respective top panels. Main effect of Drug: **** $p < 0.0001$. Unpaired t-tests: *** $p < 0.001$ pGluA1 vs. pERK1/2 for each genotype.

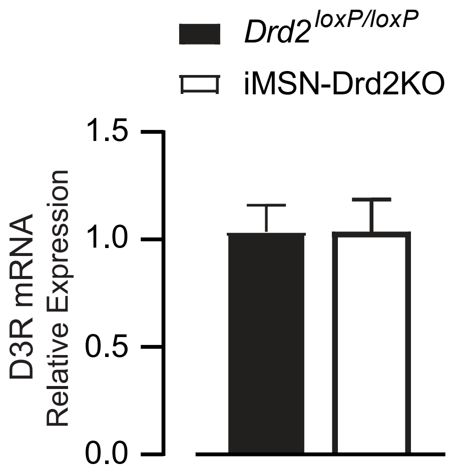

Figure S4. Downregulation of D2Rs from iMSNs does not alter striatal dopamine D3 receptor expression. The relative expression of D3 receptor mRNA within the striatum was detected using quantitative real-time PCR. Expression in iMSN-Drd2KO mice is shown compared to *Drd2*$^{loxP/loxP}$ littermate controls.

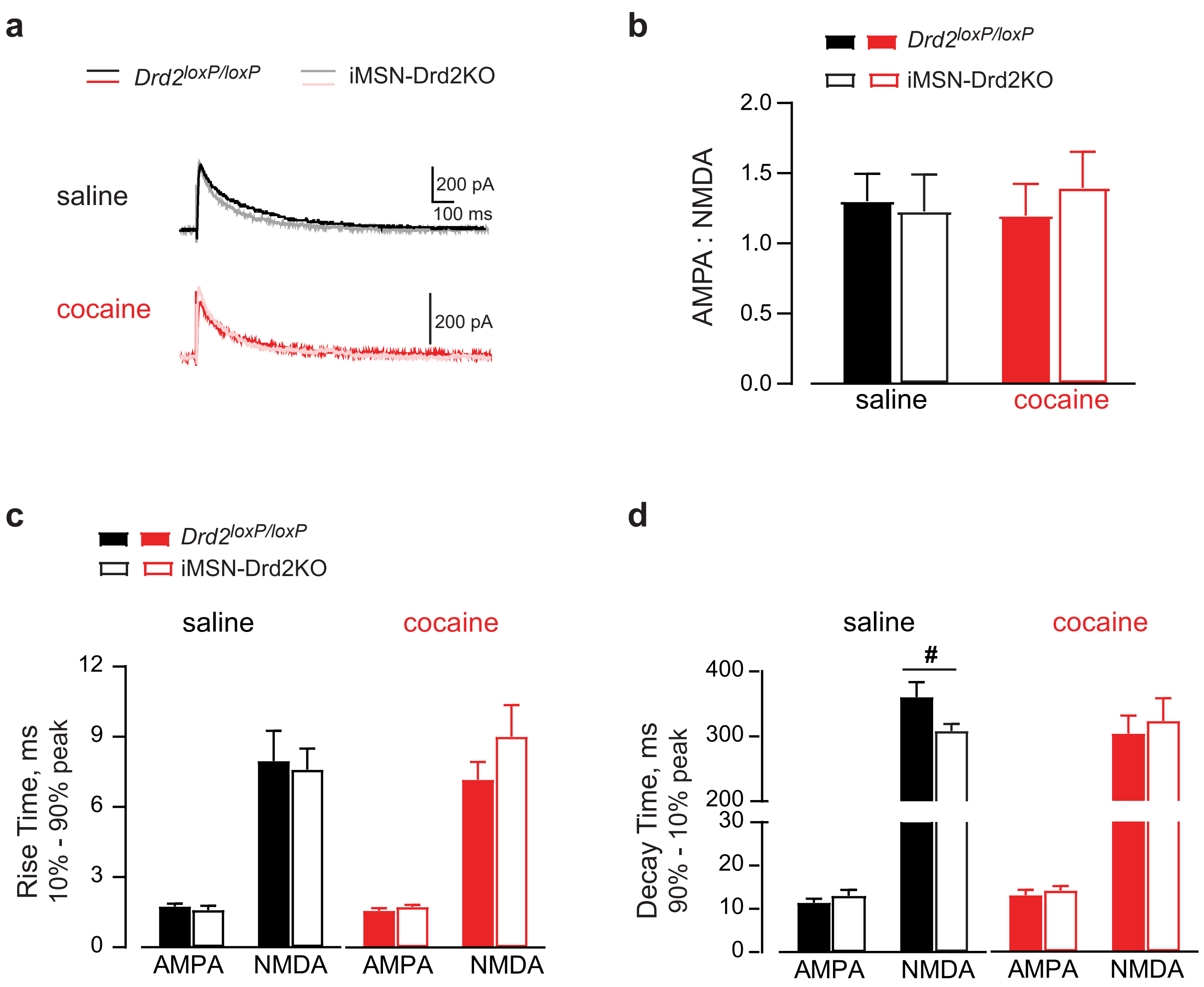

Figure S5. Similar striatal glutamate transmission in mice with low striatal D2Rs. (a) Representative electrically-evoked excitatory post-synaptic current (eEPSC) trace showing the isolated NMDA-mediated current in saline-treated (black) and cocaine-treated (red) *Drd2*$^{loxP/loxP}$ (dark) and iMSN-Drd2KO (light). Recordings were made from dMSNs in the NAc core. (b) AMPA/NMDA ratio was determined in *Drd2*$^{loxP/loxP}$ (filled) and iMSN-Drd2KO (open) mice after acute saline (black) or cocaine (15 mg/kg, red) treatment. (c,d) The rise time (c) and decay time (d) for the AMPA and NMDA mediated currents are shown in saline and cocaine treated *Drd2*$^{loxP/loxP}$ and iMSN-Drd2KO mice. Unpaired t-test: # $p = 0.07$ *Drd2*$^{loxP/loxP}$ vs. iMSN-Drd2KO.

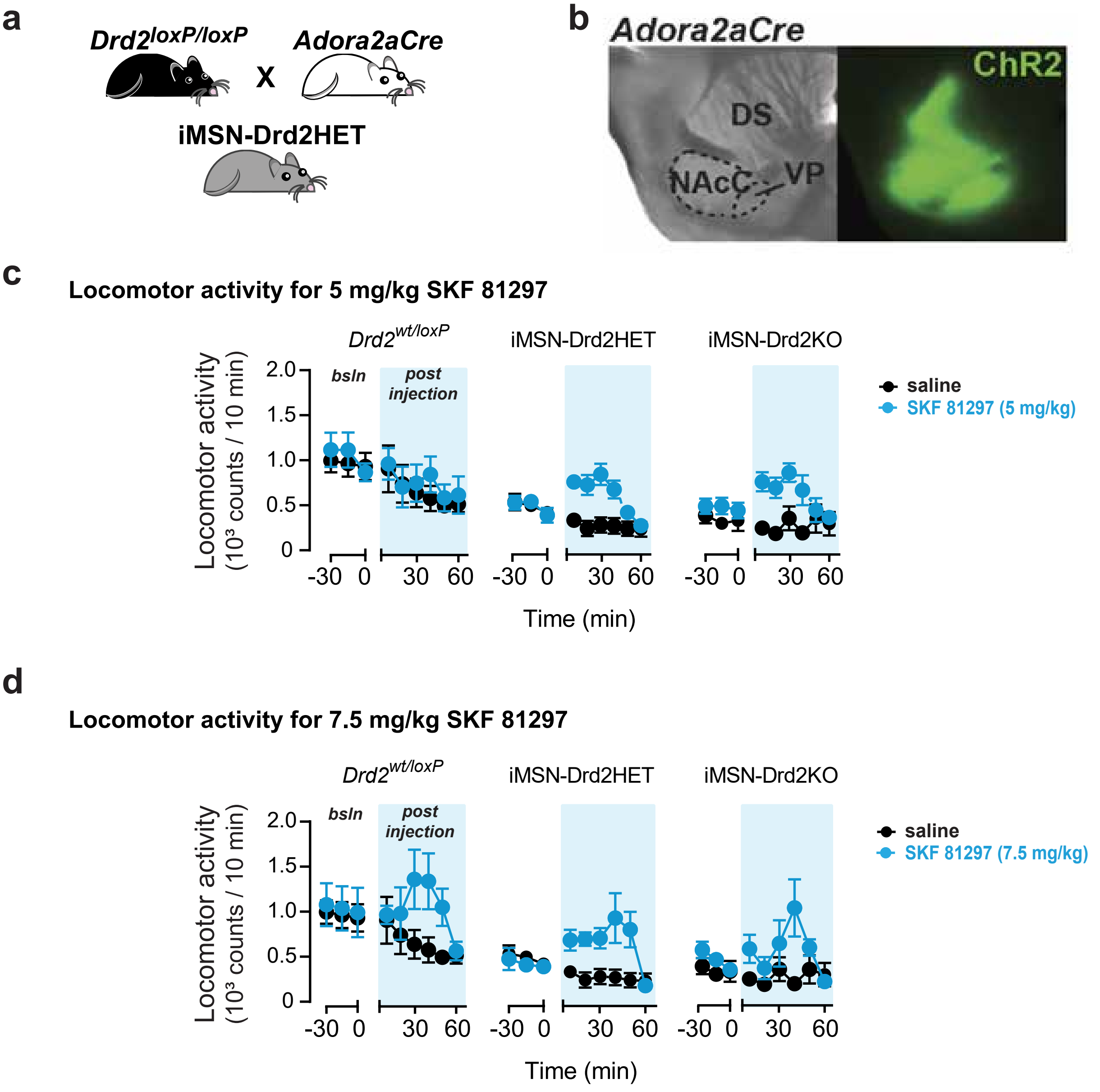

Figure S6. Mice with a partial reduction of striatal D2 receptors show enhanced locomotor response to a D1-like agonist. (a) Schematic representation of the generation of the iMSN-Drd2HET mice by crossing *Drd2*$^{loxP/loxP}$ with *Adora2aCre* mice. (b) Mice expressing Cre recombinase in iMSNs (*Adora2aCre*, iMSN-Drd2HET) showed selective, Cre-dependent expression of an AAV-ChR2-EGFP (green) in the nucleus accumbens core (NAcC). DS, dorsal striatum. VP, ventral pallidum. (c, d) Horizontal locomotor activity following injection of saline (black symbols), 5 mg/kg SKF-81297 (panel c, blue symbols), or 7.5 mg/kg SKF-81297 (panel d, blue sumbols). Locomotor activity is shown before (”bsln”) and after (”post-injection”) the saline or SKF-82197 injection (blue box) for *Drd2*$^{loxP/loxP}$ (left), iMSN-Drd2HET (middle), and iMSN-Drd2KO (right) mice.

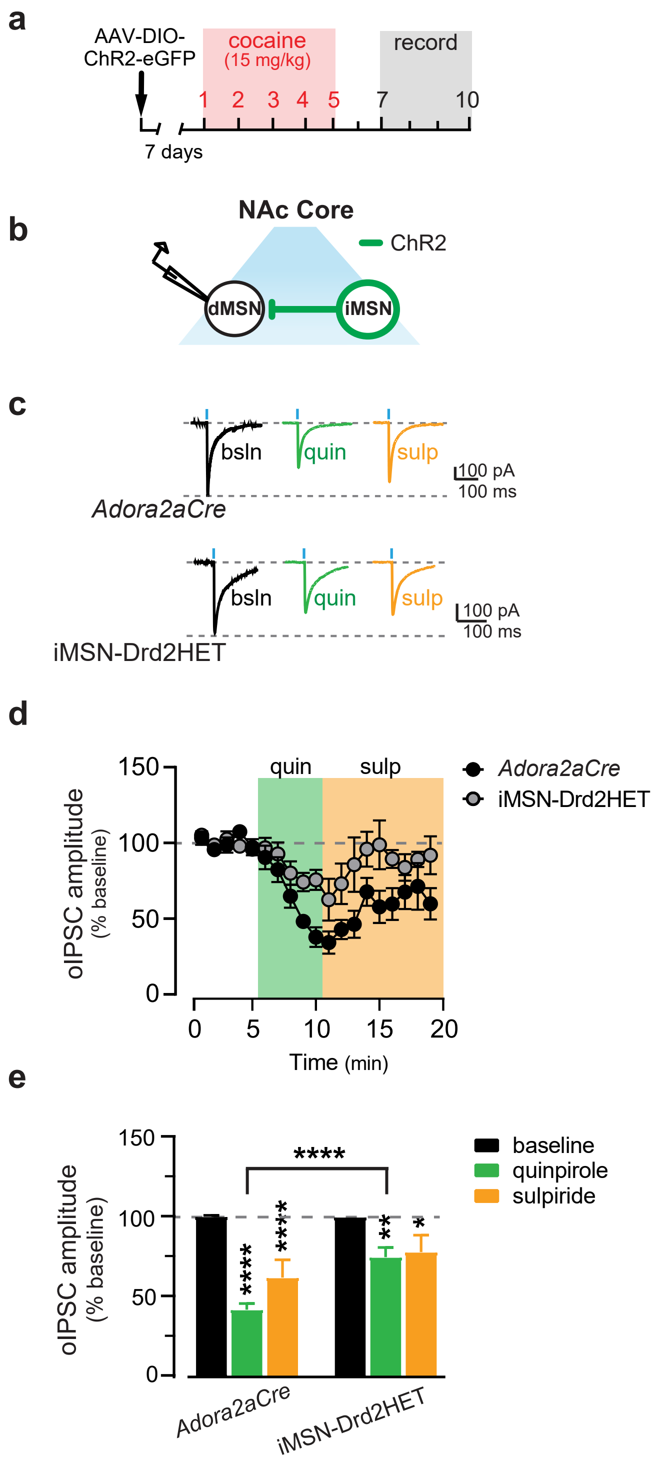

Figure S7. Repeated cocaine exposure does not downregulate striatal D2Rs in iMSNs. (a) Timeline of the experiment. (b) Schematic showing light activation of channelrhodopsin2 (ChR2)-containing iMSNs in the nucleus accumbens core (NAc Core) and whole cell recording in neighboring dMSN. (c) Representative optically-evoked inhibitory post-synaptic current (oIPSC) traces recorded at baseline (bsln), in quinpirole (1 μM), or in sulpiride (1 μM) in slices from *Adora2aCre* (top) or iMSN-Drd2HET (bottom). (d) Time course of oIPSC amplitude recorded from dMSNs during quinpirole (green) or sulpiride (blue) in *Adora2aCre* (black) or iMSN-Drd2HET (grey). (e) Average oIPSC amplitude as a percent of baseline in response to quinpirole or sulpiride in *Adora2aCre* (left) or iMSN-Drd2HET (right). Posthoc t-tests: Baseline vs. Drug: **** $p < 0.0001$, ** $p < 0.01$, * $p < 0.05$. Posthoc t-test: *Adora2aCre* vs. iMSN-Drd2HET after quinpirole treatment: **** $p < 0.0001$.

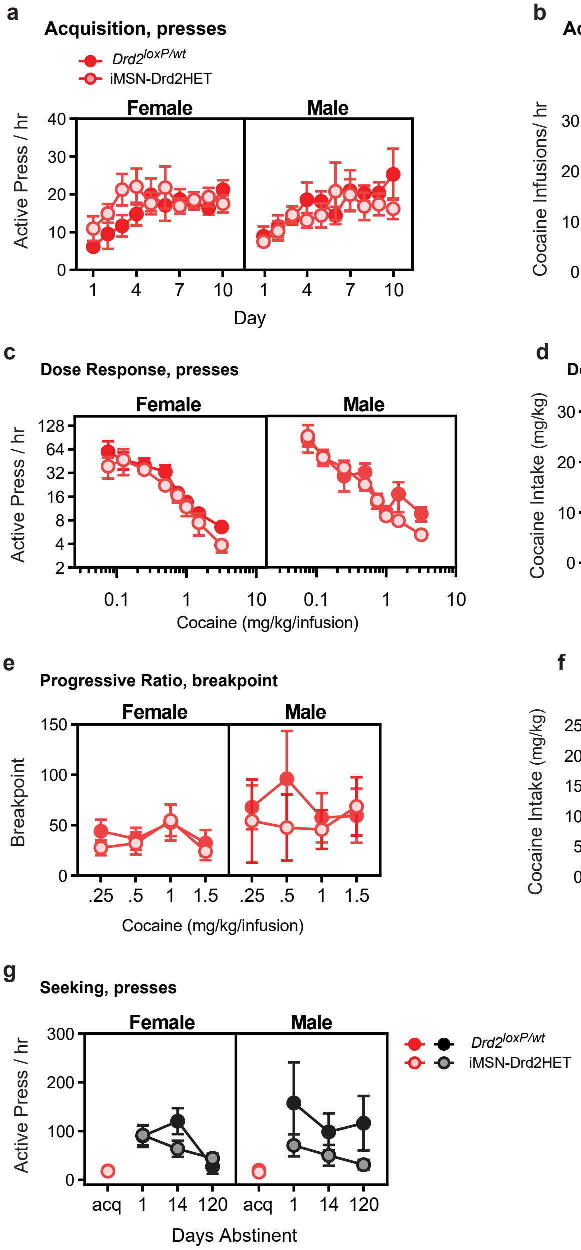

Figure S8. Cocaine self-administration and seeking is similar between sexes in mice with reduced striatal D2Rs and littermate controls (a) Rate of responding on the active lever over 10 days of acquisition (FR1, 1 mg/kg/infusion, ≤ 30 mg/kg cocaine or up to 6 h/day) for cocaine self-administration in *Drd2*$^{loxP/wt}$ (dark filled) and iMSN-Drd2HET (light filled) mice for both males and females. (b) Rate of cocaine infusions earned per hour is shown for males and females over the 10 days of acquisition. (c,d) Rate of responding on the active lever (c) and the total cocaine intake (d) is shown for males and females over increasing cocaine doses (FR1, 1 dose/hour). (e.f) The motivation for cocaine was determined by progressive ratio, wherein the response requirement for each successive cocaine infusion increased over 5 hours. The number of lever presses emitted to receive the last cocaine infusion (breakpoint, e) and the total cocaine intake (f) is shown for males and females for each cocaine dose. (g) Cocaine seeking was measured as the rate of active lever responding under extinction conditions and is shown for males and females over increasing duration of cocaine abstinence (black), and compared to the average responding over the last 4 days of acquisition (acq, red).
