# Supplementary material for: D1 receptor hypersensitivity in mice with low striatal D2 receptors facilitates select cocaine behaviors
Source: Neuropsychopharmacology. 2018 Dec 1;44(4):805–16. doi: 10.1038/s41386-018-0286-3 (PMC6372593; doi:10.1038/s41386-018-0286-3)
Supplement: Supplementary file 2 — Supplementary material [file 41386_2018_286_MOESM2_ESM.docx]

**Supplemental Materials and Methods**

*Animals*

Fluorescent identification of the *Drd1* containing striatal MSNs was accomplished using mice expressing tdTomato under the *Drd1a* promotor (Tg(Drd1a-tdTomato)6Calak, JAX016204) (Ade et al, 2011). Littermate *Drd2^loxP/wt^* or *Drd2^loxP/loxP^* or *Adora2aCre* mice were used as controls. All mice were genotyped using Transnetyx.

*Stereotaxic viral injection*

Viral injections were delivered at a rate of 100 nL/min. Stereotaxic coordinates for the NAc core were (mm from bregma): + 1.3 AP, ± 1.0 ML, -4.6 DV.

*Intra-jugular catheter placement*

The right jugular vein was isolated and a catheter was inserted through a hole in the vein made using a 27G needle. The catheter was fed subcutaneously so that the catheter inlet was fixed between the scapulae. Mice recovered for 5 – 7 days before testing began, during which time they received oral antibiotics (Trimethoprim Sulfamethoxazole, 0.72 mg/ml in drinking water). Catheter patency was tested every 7 days thereafter by administration of a ketamine-xylazine cocktail into the catheter (20 µL; 17 mg/mL and 1.7 mg/mL respectively). Mice that failed patency at any point (15% of all mice) were removed from the study.

*Quantitative polymerase chain reaction*

The probes to measure dopamine D3 receptor and beta-actin were *Drd3:* Mm00432887_m1 and *Actb*: Mm01205647_g1, respectively. Samples were run in triplicate and in parallel with negative controls. The cycling conditions were: initial hold at 95℃ (20 s), 40 cycles of 95℃ (1 s) and 60℃ (20 s).

*Western blot*

The DMS and NAc were dissected between +1.18 and + 0.74 mm anterior from bregma. The dorsal-ventral boundaries for the DMS and NAc were -2.25 to – 3.75 mm and -4.0 to -4.75 mm below brain surface, respectively. The medial-lateral boundaries for the DMS and NAc were ± 0.75 to 1.25, and ± 0.25 to 1.5 mm medial from midline, respectively. Primary antibodies used were: rabbit anti-phosphoGluA1 (Ser845, 1:500, Cell Signaling, #8084), rabbit anti-GluA1 (1:1000, Cell Signaling, # 8850), rabbit anti-pERK1/2 (1:1000, Santa Cruz, #SC7976), mouse anti-ERK (1:5000, Santa Cruz, #SC1647), and mouse anti-actin (1:10,000, Sigma, #A2228). Donkey anti-rabbit horseradish peroxidase (HRP) and donkey anti-goat HRP (1:2000) were purchased from The Jackson Laboratory.

*Immunohistochemistry*

All blocking solutions, primary and secondary antibody solutions were prepared in 0.3% TritonX-PBS (Sigma-Aldrich). The primary antibody, rabbit anti-pERK1/2, was prepared at a 1:100 dilution (Cell Signaling #4370). The secondary antibody, goat anti-rabbit Alexa 488, was prepared at a 1:500 dilution (Invitrogen, #A11034). Two sections containing the NAc core were imaged from each mouse, and three confocal images were acquired for each section. Image acquisition settings were held constant between treatment groups. The threshold for pERK fluorescence was determined by the level of background fluorescence in “no primary” control sections. The integrated density of pERK immunoreactive fluorescence in *Drd1*-expressing cells (i.e., tdTomato positive) was measured using ImageJ and expressed as density (pERK positive cells / mm^2^). The density of *Drd1*-expressing cells was measured as the number of tdTomato positive cells per square millimeter.

*Quantification of bridging collateral density*

Quantification of the density of projections from D1R-expressing MSNs was achieved using the fluorescence intensity of the genetically encoded protein tdTomato in mice expressing this transgene under the *Drd1* promoter. Fresh, serial, 150 µm thick sections were made on a vibratome and imaged using an Olympus Macroscope. Red fluorescence intensity was measured in the: globus pallidus (GP), ventral pallidum (VP), substantia nigra (SNr), dorsal striatum (DS), and nucleus accumbens (NAc). In each region, a region of equal size was drawn and the fluorescence intensity was quantified using ImageJ. The fluorescence intensity in the projection area region (GP, VP, or SNr) was normalized to the intensity in the corresponding striatal region, DS or NAc (^SNr^∕_DS,_ ^GP^∕_DS,_ and ^VP^∕_NAc_). Images of the striatal region and its corresponding projection region were taken from the same slice. Magnification was maintained between images (4x for the ^GP^∕_DS,_ and ^VP^∕_NAc_, and 1x for the ^SNr^∕_DS_).

*Operant intravenous cocaine self-administration*

Self-administration chambers (MedAssociates; 6 cm W × 17 cm L × 13 cm H) were equipped with a ventilation fan, house light, two levers (active and inactive), and drug availability light positioned above the active lever. Cocaine was delivered by a drug infusion pump (6 µl/s) via a single channel fluid swivel mounted on a counterbalanced arm (Instech). The acquisition criteria for cocaine self-administration were: 3:1 active-to-inactive lever responding and a daily cocaine intake of 30 mg/kg over the last 4 consecutive sessions. Thus, the genotypes were matched for cocaine intake history. Only data from mice that met the acquisition criteria were analyzed, and only mice that met these criteria were tested on the subsequent experiment phases (dose-response, progressive ratio and seeking).

*Electrophysiology*

Cutting solution contained (in mM): 225 sucrose, 119 NaCl, 2.5 KCl, 0.1 CaCl_2_, 4.9 MgCl_2_, 26.2 NaHCO_3_, 1 NaH_2_PO4, 1.25 glucose, and 3 kynurenic acid. Artificial cerebrospinal fluid (aCSF) was prepared as follows, (in mM): 124 NaCl, 2.5 KCl, 2.5 CaCl_2_, 1.3 MgCl_2_, 26.2 NaHCO_3_, 1 NaH_2_PO_4_, and 20 glucose. To record GABA-A receptor mediated synaptic responses, the electrode internal solution contained (in mM): 60 CsMS, 60 CsCl, 10 HEPES, 0.2 Cs-EGTA, 4 Na-ATP, 0.4 Na-GTP, and 10 phosphocreatine (pH = 7.24, ~300 mOsm). For measurements of AMPA/NMDA ratios recording electrodes were filled with internal solution containing (in mM): 120 CsMS, 10 CsCl, 10 HEPES, 4 Na-ATP, 0.4 Na-GTP, and 10 phosphocreatine-Na_2_. Minimal stimulation was used to evoke an AMPA current and was similar between genotypes (*Drd2^loxP/loxP^*: 58 ± 6.8 µA, iMSN-Drd2KO: 76 ± 15 µA).

**Supplemental Results**

*Heterozygous deletion of striatal D2Rs as a model with improved face validity*

In addition to the attenuated effect of quinpirole in iMSN-Drd2HET mice, the ability of the D2-like antagonist sulpiride to reverse the quinpirole-induced inhibition was stronger in iMSN-Drd2HET mice than in Adora2a-Cre controls (Adora2a-Cre: 25 ± 3 % inhibition, t_55_ = 2.82, *p* < 0.05; iMSN-Drd2HET: 6 ± 13 % inhibition, t_55_ = 0.70, *p* > 0.05; Figure 4).

*No further downregulation of D2Rs in iMSNs upon repeated cocaine exposure*

Reports in the literature indicate that repeated treatment with cocaine can lower D2R availability in the striatum (Nader et al, 2006). We hypothesized that mice with a pre-existing low level of D2Rs in iMSNs would be more susceptible to further downregulation of D2Rs by repeated cocaine exposure. To test this, we treated iMSN-Drd2HET and Adora2aCre controls with cocaine for five consecutive days and measured the efficacy of quinpirole to inhibit collateral GABA transmission in the slice (Figure S7). Similar to saline-treated iMSN-Drd2HET mice from our previous experiment, quinpirole produced a 25 ± 6 % inhibition of collateral GABA transmission in cocaine pre-treated iMSN-Drd2HET mice relative to baseline (2-way ANOVA; Drug x Genotype interaction: F_2,43_ = 6.7, *p* < 0.01; n = 9-10 cells/genotype; t_43_ = 3.5, *p* < 0.01). Quinpirole also induced a 58 ± 4 % inhibition of collateral GABA transmission in cocaine pre-treated Adora2aCre controls (t_43_ = 8.6, *p* < 0.0001), which was significantly greater than the magnitude of quinpirole’s effect in the iMSN-Drd2HET mice (t_43_ = 4.7, *p* < 0.0001). These data indicate that repeated cocaine treatment does not functionally downregulate D2Rs in iMSNs. Additionally, iMSN-Drd2HET mice do not appear to be differentially sensitive to the effect of repeated cocaine on the ability of D2Rs expressed in iMSNs to restrain collateral GABA transmission.

*Cocaine seeking and taking in iMSN-Drd2HET mice*

Analysis of the inactive lever responding across all self-administration procedures was performed to examine possible genotype differences on the overall motor behavior during these tests. We found no consistent differences in inactive lever responding in most stages tested, except for the first week of acquisition and at low cocaine doses during the dose-response. During acquisition, iMSN-Drd2HET mice showed a higher mean rate of inactive lever responding, which was primarily driven by a higher rate of inactive responding during the first week (5.8 ± 0.9 and 3.1 ± 0.4 for iMSN-Drd2HET vs. Drd2^loxP/loxP^, respectively; 2-way RMANOVA: Main effect of genotype: F_1,299_ = 7.925, p < 0.01).

Analysis of the inactive lever responding during the dose-response revealed that inactive lever activity increased as the cocaine unit dose decreased for both genotypes, but that iMSN-Drd2HET mice showed less activity low doses of cocaine (2-way ANOVA: Main effect of dose: F_7,199_ = 3.17, p < 0.001; Main effect of genotype: F_1,199_ = 5.90, p < 0.01). Importantly, though, inactive responding was similar between genotypes at the highest unit dose, 3.2 mg/kg/infusion, which is the dose at which intake was lower for iMSN-Drd2HET mice (0.67 ± 0.53 and 1.42 ± 0.71 inactive presses/hour for Drd2^loxP/loxP^ and iMSN-Drd2HET, respectively). Thus, a general motor impairment is not likely driving the decreased intake of high-dose cocaine in iMSN-Drd2HET mice.

While there were no significant differences between genotypes in the breakpoint for cocaine, there were differences in the rate of active lever pressing during the progressive ratio test (2-way ANOVA; Main effect of Genotype: F_1,96_ = 4.65, *p* < 0.05). iMSN-Drd2HET mice showed a lower average rate of active lever pressing across all cocaine doses during the progressive ratio test (*Drd2^loxP/wt^* : 90.1 ± 13.7, iMSN-Drd2HET: 54.9 ± 2.3 presses/h), but there were no differences between genotypes in the rate of inactive lever pressing. One interpretation of this is that iMSN-Drd2HET mice have a mild motor deficit that is only evident when the operant requirement is pushed during progressive ratio sessions.

While the iMSN-Drd2HET mice showed suppressed seeking during abstinence, this effect did not appear due them being generally less active. Analysis of the inactive lever responding showed that both iMSN-Drd2HET mice and littermate controls increased their inactive lever responding on the first seeking day, and then gradually decreased inactive responding over progressive abstinence (2-way ANOVA: Main effect of abstinence: F_3, 96_ = 12.14, p < 0.0001). Interestingly, neither genotype showed a progressive enhancement in seeking behavior with increased abstinence, as has been reported in previous literature (Grimm et al, 2001; Li et al, 2016). Our finding, however, is in agreement with recent data in mice showing that incubation of craving only occurs when mice are initially trained on a higher unit dose of cocaine (1.5 mg/kg; Terrier *et al*, 2015).

**Supplemental References**

Terrier J, Luscher C, Pascoli V, Lüscher C, Pascoli V (2015). Cell-Type Specific Insertion of GluA2-Lacking AMPARs with Cocaine Exposure Leading to Sensitization, Cue-Induced Seeking, and Incubation of Craving. *Neuropsychopharmacology* **41**: 1779–89.
